# Potential Beneficial Effects of Hydroxyapatite Nanoparticles on Caries Lesions In Vitro—A Review of the Literature

**DOI:** 10.3390/dj11020040

**Published:** 2023-02-07

**Authors:** Eisha Imran, Paul R. Cooper, Jithendra Ratnayake, Manikandan Ekambaram, May Lei Mei

**Affiliations:** 1 Department of Dental Materials, Islamabad Medical and Dental College, Islamabad 44000, Pakistan; 2 Sir John Walsh Research Institute, Faculty of Dentistry, University of Otago, Dunedin 9016, New Zealand

**Keywords:** in vitro, hydroxyapatite nanoparticles, mineralisation, antimicrobial, caries

## Abstract

Dental caries is one of the most common human diseases which can occur in both primary and permanent dentitions throughout the life of an individual. Hydroxyapatite is the major inorganic component of human teeth, consequently, nanosized hydroxyapatite (nHAP) has recently attracted researchers’ attention due to its unique properties and potential for caries management. This article provides a contemporary review of the potential beneficial effects of nHAP on caries lesions demonstrated in in vitro studies. Data showed that nHAP has potential to promote mineralization in initial caries, by being incorporated into the porous tooth structure, which resulted from the caries process, and subsequently increased mineral content and hardness. Notably, it is the particle size of nHAP which plays an important role in the mineralization process. Antimicrobial effects of nHAP can also be achieved by metal substitution in nHAP. Dual action property (mineralizing and antimicrobial) and enhanced chemical stability and bioactivity of nHAP can potentially be obtained using metal-substituted fluorhydroxyapatite nanoparticles. This provides a promising synergistic strategy which should be explored in further clinical research to enable the development of dental therapeutics for use in the treatment and management of caries.

## 1. Introduction

Dental caries is one of the most prevalent and ubiquitous non-communicable diseases worldwide. It is characterised by the progression of demineralization of the dental hard tissue, resulting in an imbalance in the equilibrium between the tooth mineral and biofilm [1]. Previously, the management of caries was driven by the understanding that it was a purely infectious disease and could only be managed invasively by removing all demineralized tissue. However, contemporary treatment strategies to manage caries have changed from the traditional surgical approach to a medical model, which now often includes dietary analysis and advice, oral hygiene instruction, placement of fissure sealants, and mineralization control by topical application of fluoride or other mineralising agents [2]. Among the available mineralization control strategies, fluoride-based treatments have the highest level of supporting evidence. Their widespread use is generally considered the main reason for reducing the incidence of dental caries in most populations. The released fluoride prevents enamel demineralization and reduces caries susceptibility by its incorporation into enamel hydroxyapatite, therefore, stabilizing the crystal structure by forming fluorapatite and lowering the solubility product constant (Ksp) [3]. Consequently, this increases resistance to acid demineralization. However, currently available fluoride therapies have limited efficacy in some individuals, and at the population level, the effect of fluoride in reducing dental caries prevalence is reaching a plateau [4]. More recently, investigators have been developing new mineralization approaches to close this gap in efficacy. Calcium and phosphate-based treatment systems are designed to ensure a constant supply of these ions and aim to directly increase mineral concentration in the environment around the caries lesions [5]. These systems include the use of amorphous calcium phosphate (ACP), functionalized tricalcium phosphate (fTCP), bioactive glass containing calcium sodium phosphosilicate and hydroxyapatite nanoparticles (nHAP) [6].

The enamel rod (prism) is the basic unit of human tooth enamel. Enamel rods are tightly packed hydroxyapatite (HAP) crystals structures which are hexagonal in shape, with sizes up to 70 nm in width and 30 nm in thickness. They provide rigidity to the enamel rod and provide significant strengthen to the enamel [7]. HAP is a mineral form of calcium apatite with the molecular formula Ca_10_(PO_4_)_6_(OH)_2_ and a calcium-to-phosphorus molar ratio of 1:67. It has a crystal lattice and its chemical composition is most similar to the apatite crystals of the human enamel. HAP also exhibits good biocompatibility and bioactivity [8]. Nanoparticles have a large surface area to volume ratio, which results in them exhibiting different biomedical activities compared with materials of increased size, furthermore, they can possess unique physical properties that make them desirable for use in biology and material science [9]. The synthesis of hydroxyapatite as nanosized particles has the potential to enable an improved physical and chemical characteristic by the enlargement of the reactive surface area. With respect to morphology and structure, the crystal lattice of nHAP is predicted to resemble the tooth enamel better and consequently would have beneficial effects for hard tissues repair.

Although there are a few previous reviews reporting nHAP in dentistry, they are either not contemporary [10] nor discuss its use within a broad scope of dental application [11,12]. One published systematic review reports on the efficacy of nHAP in caries prevention, however, no strong conclusions were made due to only a very limited number of in vivo and in situ studies being evaluated [13]. A significant number of in vitro studies have now investigated the effect of nHAP on dental hard tissues and cariogenic bacteria and biofilms have been conducted. Consequently, this review aims to provide recent novel information and reports on the action of nHAP in caries management, focussing on its effects on the dental hard tissues and cariogenic bacteria.

## 2. Materials and Methods

### 2.1. Literature Search Strategy

A literature search was undertaken in PubMed to identify English language publications from 2010 to 2022. The keywords included: (nano-HAP OR HAP-NPs OR nano-hydroxyapatite OR nanohydroxyapatite OR hydroxyapatite OR Hydroxyapatite nanoparticles) AND (caries OR carious OR cariogenic OR tooth decay).

### 2.2. Study Inclusion and Exclusion Criteria

#### 2.2.1. Inclusion Criteria

(1) Laboratory studies.
(2) Studies related to antimicrobial or biocompatibility effect of nHAP on cariogenic species.
(3) Studies related to the mineralization effect of nHAP on tooth hard tissue (enamel, dentine, cementum) (this includes remineralization and modulation of demineralization on hard tissue).

#### 2.2.2. Exclusion Criteria

(1) Studies not reported using the English language.
(2) Studies on HAP that were not in the nano-scale range.
(3) Studies not related to dental caries and abstracts without the associated full papers.
(4) Case reports, conference papers, book chapters, patents, letters to the editor, systematic reviews, meta-analyses, and literature review papers.

## 3. Results

**Figure 1:**
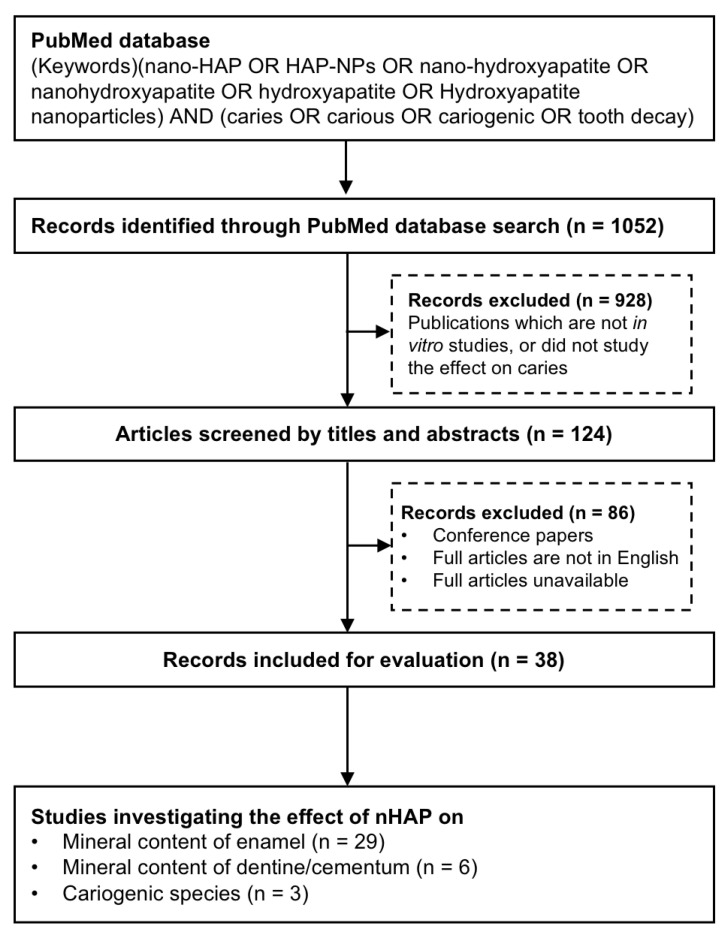
Flowchart of study selection process for final review.

A total of 1052 potentially eligible articles published up to February 2022 were identified using the search terms described (Figure 1). After screening titles and abstracts, 125 articles remained for further analysis. Full-text reading was undertaken and 38 articles were finalized for inclusion in this review. Among these articles, twenty-nine studies investigated the effects of nHAP on enamel mineralization (Table 1), five studies were reported on dentine remineralization and one study on cementum (Table 2), and three studies investigated the antimicrobial effect of nHAP on cariogenic bacteria (Table 3). Twenty-two studies used toothpaste containing nHAP, nine studies used an aqueous slurry of nHAP; nHAP was also incorporated in other formats such as tooth cream, tooth gel, and resin infiltration in three studies.

### 3.1. Effect of nHAP on Mineralization of Enamel

Table 1 presents the main findings of the studies which investigated the role of nHAP on enamel mineralization. Enamel specimens were prepared using either bovine or human teeth; the majority (28 out of 29) of studies generated early artificial caries lesions by chemical means on enamel specimens prior to treatment to mimic incipient enamel caries. These caries lesions were then treated with a range of treatments including nHAP formulations. The format of the nHAP containing products included toothpastes (18 studies) [14,15,16,17,18,19,20,21,22,23,24,25,26,27,28,29,30,31], slurries prepared with nHAP powder (eight studies) [26,32,33,34,35,36,37,38], resin infiltration (one study) [39], cream (one study) [40], and gel (one study) [41]. Nineteen studies employed microhardness to evaluate the effect of nHAP on remineralization. Eighteen of these studies found that products with a nHAP component demonstrated increased enamel surface microhardness when compared with control groups. Five studies measured the mineral recovery from the enamel lesion after nHAP treatment, with all studies reporting that the nHAP treatment groups exhibited higher mineral recovery when compared with negative controls. Other studies also demonstrated the repair of surface defects and a smoother uniform distribution of Ca/P particles in the surface area exposed to nHAP when compared with other treatment groups [14,15,16]. A few studies reported nHAP used in combination with fluoride revealed a significant reduction in lesion depth in contrast to other agents [16,17].

### 3.2. Effect of nHAP on Mineralization of Dentine/Cementum

Table 2 presents the key findings from the six studies which investigated the role of nHAP on dentine or cementum mineralization, with inconsistent findings being reported. The format of the nHAP products used were toothpaste (four studies) [19,20,42,43], slurry prepared using nHAP powder (one study) [44], and gel (one study) [41]. One study identified a significant increase in surface microhardness following nHAP treatment of cementum when compared with fluoride treatment [41]. While another study demonstrated formation of a partially filled remineralized layer after nHAP treatment in contrast to the group treated with 30% pre-reacted glass ionomer (S-PRG) filler, which resulted in formation of a fully remineralized layer on dentine [42]. Similarly, a further study showed reduction in lesion depth when nHAP was used in combination with fluoride [18]. Notably, one study claimed that nHAP was unable to reduce microhardness loss [19].

### 3.3. Effect of Hydroxyapatite Nanoparticles on Cariogenic Bacteria

Table 3 presents the key findings of studies which investigated the effect of nHAP on cariogenic bacteria and biofilms. One study showed that pure nHAP tends to enhance *Streptococcus mutans* biofilm formation; the mechanisms behind could be that nHAP was capable to increase glucosyltransferase transcription which resulted in an increase in production of insoluble glucans [45]. However, there were different results when calcium ions were substituted partially with metal ions. A study investigated the effect of loading zinc ions with nHAP in combination with alendronate grafted poly-acrylic acid, concluding that zinc ions significantly enhanced the materials’ antibacterial effect against *S. mutans* in contrast to samples where nHAP was used alone [32]. In another study, toothpaste with zinc substituted nHAP was also found to be less antimicrobial against *S. mutans* when compared to the one with strontium (Sr), magnesium (Mg), and fluoride (F) substituted nHAP [46].

**Table 1:** Summary of the effect of hydroxyapatite nanoparticles on enamel mineralization.

Reference	Experimental Groups	Study Model and Design	nHAP Details	Main Findings
Huang et al. 2010 [33]	Sodium fluoride (positive control) Deionized water (negative control) Galla chinensis (a traditional Chinese medicine) nHAP nHAP + Galla chinensis	Substrate: Enamel specimens were prepared from bovine incisors; Early artificial carious lesions were formed before treatment. Model: pH-cycling for 12 days, treatment 4 × 3 min daily. Assessments: Mineral loss and surface morphology by surface microhardness, polarized light microscopy, X-ray diffraction, and scanning electron microscopy.	Particle size: 5–26.7 nm (diameter), 30–84 nm (length). Format: Aqueous slurry in distilled water prepared from purchased powder.	nHAP showed increased surface hardness compared with negative control. nHAP enabled mineral deposition predominately in the outer layer of the lesion and showed limited capacity to reduce lesion depth.
Huang et al. 2011 [34]	micro-HAP (pH 7) nHAP (pH 7) nHAP (pH 4) nHAP (pH 5.5) Distilled and deionized water1 (pH 7) (negative control) Equivalent test solution (pH 7) (positive control, same concentration of free ion in the nanoHA group at equilibrium, pH 7.0) Distilled and deionized water (pH 7) (negative control) (a common control used between the original and new experiment)	Substrate: Enamel specimens were prepared from bovine incisors; Early artificial carious lesions were formed before treatment. Model: pH cycling for 12 days, Treatment: 4 × 3 min daily Assessments: Mineral loss by surface microhardness and cross-sectional microhardness analysis, polarized light microscopy examination.	Particle size: Length: 60–80 nm Diameter: 10–20 nm, The surface area of nHAP = 86.2 m^2^/g and micro HAP = 12.5 m^2^/g Format: Aqueous slurry in distilled water prepared from purchased powder.	nHAP demonstrated mineral deposition in the outer layer of the lesion and showed negligible potential in reducing the depth of the lesion. nHAP with lower pH showed higher surface microhardness recovery.
Tschoppe et al. 2011 [20]	1.5 mM Calcium and phosphate 20 wt% Zinc carbonate-nHAP 24 wt% Zinc carbonate-nHAP 0.14 wt% Aminefluoride 7 wt% nHAP.	Substrate: Enamel specimens were prepared from bovine incisors; Early artificial carious lesions were formed before treatment. Model: Remineralizing solution for 2 and 5 weeks. Treatment: 2 × 2 min daily Assessment: Mineral loss and lesion depth by transverse microradiography	Particle size: 20 nm in size, with granular dimensions up to 100–150 nm. Format: Commercially available nHAP in paste form (BioRepair and BioRepair Sensitive; Dr. Kurt Wolff: Bielefeld, Germany) were obtained and diluted to form a slurry homogenous mixture.	7% nHAP toothpaste demonstrated less mineral loss on enamel lesions in contrast to 0.14% Amineflouride. The surface layer of the lesion showed remineralization.
Swarup et al. 2012 [35]	10% nHAP 2% Sodium fluoride	Substrate: Enamel specimens were prepared from human premolars; Early artificial carious lesions were formed before treatment. Model: Artificial saliva for 10 days treatment: One-off treatment Assessment: Mineral recovery by surface micro hardness analysis; surface morphology and mineral content by SEM-EDX.	Particle size: length: 50–100 nm, width: 20–40 nm. Format: Aqueous slurry in distilled water prepared from purchased powder.	A significant higher surface mineral recovery content was observed in the specimens when treated with nHAP in contrast to sodium fluoride. Crystal nucleation in the demineralized pores along with the formation of a uniform thick layer was observed when the specimens were exposed to nHAP.
Comar et al., 2013 [19]	20% nHAP 20% nHAP + 0.2% NaF 10% nHAP 10% nHAP + 0.2% NaF Placebo paste (without fluoride and nHAP) 0.2% NaF CPP-ACP CPP-ACP + 0.2% NaF	Substrate: Enamel specimens were prepared from labial surface of the bovine crown. Model: pH cycling for 7 days. Treatment: Brushed for 1 min, twice a daily. Assessment: Surface microhardness by Knoop hardness tests.	Particle size: experimental paste consisted of Calcium Phosphate (100 nm). Format: paste form.	nHAP treatment was unable to reduce the loss of enamel surface hardness regardless of fluoride addition.
Mielczarek 2014 [21]	nHAPF group (1% nHAP + 1450 ppm NaF) Fluoride group: (1450 ppm NaF) Placebo group (distilled water)	Substrate: Enamel specimens were prepared from human teeth; Early artificial carious lesions were formed before treatment. Model: pH cycling for 3 weeks. Treatment: 2 × 2 min daily Assessment: Surface microhardness by Vickers method; Surface roughness (Ra value) by profilometer.	Particle size: not reported. Format: nHAPF: (1% nHAP and 1450 ppm NaF) was obtained in toothpaste form and diluted with water in the ratio 1:3 to form a dentifrice slurry.	nHAPF group revealed significant increase in surface microhardness as compared to the fluoride group. Surface roughness significant decrease in the nHAPF group as compared with the Fluoride group.
Haghgoo et al. 2014 [22]	Distilled water (Negative control) 2% nHAP 5% nHAP 10% nHAP 0.2% NaF	Substrate: Enamel specimens were prepared from human premolars; Early artificial carious lesions were formed before treatment. Model: Pre-immersed in artificial saliva for 12 h before treatment. Treatment: The specimens were immersed into distilled water plus different treatment for 12 h. Assessment: Microhardness test.	Particle size: not reported Format: Commercial nano HAP was obtained and mixed into toothpaste.	No significant difference was observed in hardness values after treatment.
de Carvalho et al. 2014 [23]	No treatment (Negative control) Fluoride varnish nHAP paste CPP-ACP paste	Substrate: Enamel specimens were prepared from human third molars; Early artificial carious lesions were formed before treatment. Model: pH cycling for 7 days. Treatment: 5 min per day for tooth paste groups, one-off treatment for varnish. Assessment: Surface microhardness test by Knoop hardness test, surface morphology by AFM.	Particle size: not reported. Format: nHAP was commercially obtained from Desensibilize Nano P (FGM Produtos Odontol gicos, Joinville, Brazil) in toothpaste form.	Surface microhardness of the specimens treated by nHAP are higher than other groups. AFM analysis revealed formation of a globular layer on the surface after application of nHAP.
Vyavhare et al. 2015 [24]	10% nHAP 10% CPP-ACP 1000 ppm sodium fluoride Deionized water (Negative control)	Substrate: Enamel specimens were prepared from human permanent maxillary incisor; Early artificial carious lesions were formed before treatment. Model: pH cycling for 12 days. Treatment: pastes were applied by an applicator brush and left undisturbed for 3 min. Frequency not mentioned. Assessment: Surface microhardness by Vickers hardness test and SEM (Scanning electron microscope).	Particle size: crystal size: 20–40 nm. Format: 10% in toothpaste form.	nHAP group demonstrated significant increase in microhardness recovery when compared with CPP-ACP and negative control. No significant difference was obtained when compared with the sodium fluoride treatment group.
Haghgoo et al. 2016 [25]	Novamin (Synthetic mineral compound consisting of Calcium Sodium Phosphorus and Silica) nHAP	Substrate: Enamel specimens were prepared from human deciduous anterior teeth; Early artificial carious lesions were formed. Model: pH cycling for 5 days. Treatment: 2 × 2 min daily Assessment: Mineral changes by surface micro-hardness.	Particle size: not reported. Format: nHAP toothpaste.	No statistically significant difference in the surface microhardness between the Novamin group and nHAP group.
Krishnan et al., 2016 [26]	No treatment (Negative control) ACP-CPP nHAP (commercially prepared) nHAP (custom made) 25 mol% Sr doped nHAP 50 mol% Sr doped nHAP	Substrate: Enamel specimens were prepared from human premolars; early artificial carious lesions were formed before treatment. Model: nHAP was mixed with remineralizing solution during a 21 days pH cycling. Assessment: Toxicity analysis using Cell viability assay (MTT), surface topography by AFM and SEM, crystallisation by XRD, surface microhardness by micro-indentation testing.	Particle size: commercially obtained (Acclaim, group pharmaceuticals limited) Wet chemical method was used for the synthesis of nHAP powder, particle size was <125 µm. Sr doped HAP was synthesized similarly by the co-precipitation method. Format: pastes or powder.	Commercially and custom prepared nHAP demonstrated toxic effects on cell viability. The capacity to agglomerate was best observed in 50% Sr doped nHAP. 25% Sr demonstrated highest crystallinity along with reduced size of the particles. Enhanced surface roughness in Sr doped samples. Highest hardness values in 50% Sr doped nHAP treated group.
Andrade Neto et al. 2016 [39]	No infiltration treatment (Control 1) Resin infiltrates (Control 2) nHAP 0 h (no hydrothermal treatment) nHAP 2 h (infiltrate in combination with nHAP after 2 h hydrothermal treatment) nHAP 5 h (infiltrate in combination with nHAP after 5 h hydrothermal treatment)	Substrate: Enamel specimens were prepared from human premolars; Early artificial carious lesions were formed before treatment. Model: pH cycling for 4 days Treatment: remineralization sol for 20 h. Assessment: crystallisation by XRD, FTIR and TEM; Surface microhardness by Knoop microhardness test.	Particle size/shape: Hexagonal in shape and possessed rod-shaped morphology were prepared using a hydrothermal method. Format: nHAP was incorporated in the resin infiltrates.	Micro-hardness tests revealed higher enamel resistance of nHAP-2h and nHAP-5h against demineralization compared to control nHAP-free and Hap-0 h infiltration. Enamel from two nHAP + resin infiltrate groups demonstrated higher crystallinity than control groups.
Ebadifar et al. 2017 [27]	nHAP toothpaste (7% nHAP + 1000 ppm fluoride) Fluoride toothpaste (1000 ppm)	Substrate: Human enamel specimens from premolars. Early artificial carious lesions were formed before treatment. Model: pH cycling. Treatment: 15 s twice daily for 15 days. Assessment: Vickers hardness test.	Particle size: not reported. Format: Sol-gel technique was used to prepare nHAP and was added to the toothpaste in 7% concentration.	nHAP treated group showed significant increase in hardness values.
Kamath et al. 2017 [40]	f-TCP (TCP + 0.21% *w/w* NaF) Fluoridated dentifrice (1000 ppm F) CPP-ACPF ACPF (CPP-ACP + 900 ppm fluoride) nHAP + 1450 ppm NaF	Substrate: Enamel specimens were prepared from human primary teeth; Early artificial carious lesions were formed before treatment. Model: 14 days in artificial saliva. Treatment: 3 min daily. Assessment: Laser fluorescence signal by DIAGNOdent, morphology, and mineral content by SEM-EDX.	Particle size: not reported. Format: Commercially available nHAP was obtained from Reminpro. It is a water-based cream containing hydroxapatite and fluoride (1450 ppm sodium fluoride).	All groups showed increased mineral content after treatment. No difference was detected regarding calcium and phosphate levels between the groups.
Sharma et al. 2017 [28]	nHAP toothpaste CPP-ACP toothpaste	Substrate: Enamel specimens were prepared from human premolars; Early artificial carious lesions were formed before treatment. Model: artificial saliva for 15 and 30 days. Treatment: brushing 2 min daily. Assessment: Surface hardness and mineral content by Vickers hardness test and SEM-EDX.	Particle size: not reported. Format: nHAP toothpaste was commercially obtained from ReminPro, VOCO.	nHAP is more effective as compared to CPP-ACP, in increasing the calcium and phosphorus content of enamel, and this effect is more evident over a longer treatment period. Microhardness values were not significantly different between the groups.
Daas et al. 2017 [18]	No treatment (Control) Fluoride varnish (5% NaF, 22,600 ppm fluoride ion) nHAP toothpaste	Substrate: Enamel specimens were prepared from human premolars; Early artificial carious lesions were formed before treatment. Model: pH cycling for 7 days. Treatment: Brushed for 10 s and left for 4 min daily for toothpaste, one-off application for varnish. Assessment: Laser fluorescence signal by DIAGNOdent, morphology by SEM.	Particle size: not reported. Format: nHAP was commercially obtained as toothpaste (Desensibilize Nano P, FGM Produtos Odontol gicos, Joinville, Brazil).	No significant difference in terms of DIAGNOdent reading between nHAP and fluoride varnish group. SEM images revealed a smoother surface in nHAP group in-contrast to Fluoride varnish.
Alsherif et al. 2017 [36]	Artificial saliva (Control 1) Artificial caries + artificial saliva (Control 2) APF gel 10% nHAP	Substrate: Enamel specimens were prepared from human premolars; Early artificial carious lesions were formed before treatment. Model: artificial saliva for 10 days. Treatment: 10 min daily. Assessment: mineral content by polarized light microscope, surface morphology by SEM and surface microhardness by Vickers hardness test.	Particle size: Dimensions <200 nm Format: nHAP powder was commercially obtained to form nHAP slurry.	Polarized light microscope analysis revealed mineral recovery in nHAP group, and this was higher than that in the APF group. nHAP group revealed an apatite layer, covering the demineralized pores. There was no difference in microhardness recovery between nHAP and APF groups.
Talaat et al. 2018 [16]	nHAP (Calcium nano phosphate paste) CPP-ACPF Control (no treatment)	Substrate: Enamel specimens were prepared from human premolars; Early artificial carious lesions were formed before treatment. Model: artificial saliva for 7 days. Treatment: 5 min daily. Assessment: Surface micro-hardness test and surface morphology by SEM.	Particle size: not reported. Format: Calcium nano phosphate paste (Desensibilize Nano P, FGM Produtos Odontol gicos, Joinville, Brazil) which is Calcium nanophosphate organized in a crystalline form of hydroxyapatite, it also contains potassium nitrate, water, surfactant, tensoactive, flavor, 9000 ppm sodium fluoride.	Surface micro-hardness test revealed same results in groups of nHAP and CPP-ACP with improvement in surface defects of demineralized enamel.
Juntavee et al., 2018 [41]	nHAP gel 0.21% NaF: (Clinpro) Control (deionized water)	Substrate: Enamel specimens were prepared from human mandibular third molars; Early artificial carious lesions were formed before treatment. Model: Remineralized gels for 30 days. Treatment: 2 × 4 min daily. Assessment: Surface microhardness by Vickers Microhardness, surface morphology by SEM.	Particle size: not reported. Format: Gel form of nHAP was obtained (10% nano-hydroxymethyl cellulose).	Significantly higher surface microhardness was revealed in nHAP group in contrast to NaF and control groups. SEM revealed NHA particle deposition in the area of remineralization after nHAP treatment.
Madhusudanan et al. 2018 [29]	Sound enamel + no treatment (Negative Control 1) Demineralized enamel + no treatment (Negative Control 2) Potassium nitrate containing toothpaste CPP-ACP containing toothpaste nHAP containing toothpaste	Substrate: Enamel specimens were prepared from human premolars; Early artificial carious lesions were formed before treatment. Model: 10 days in artificial saliva. Treatment: 2 × 3 min daily. Assessment: Surface microhardness by Vickers hardness test.	Particle size: not reported. Format: 1% nano HAP was obtained from Aclaim group, India (toothpaste).	nHAP treated enamel lesions demonstrated highest surface microhardness in comparison with other groups.
Memarpour et al. 2019 [37]	Etchant and sealant Etchant, 0.15% nHAP and sealant Etchant, 0.03% nHAP and sealant Etchant, 0.15% nHAP+ 0.05% Sodium Hexametaphosphate, and sealant Etchant, 0.03% nHAP+ 0.01% Sodium Hexametaphosphate, and sealant	Substrate: Enamel specimens were prepared from human 3rd molars teeth; Early artificial carious lesions were formed before treatment. Model: a thermocycling at temperatures between 5 °C and 55 °C for 1000 cycles. Treatment: after etchant, either nHAP or nHAP + Sodium Hexametaphosphate were applied for 5 min at slow speed rotation, the teeth were then dried and sealants were applied. Assessment: bonding strength by micro-leakage and shear bond strength, surface morphology by SEM, mineral content by SEM-EDX.	Particle size: average diameter 10.67 nm. Format: nHAP powder diluted in distilled water and ≥ 99.5% acetone to prepare the solutions.	The levels of Ca and P demonstrated a non-significant difference between the groups treated with nHAP. nHAP 0.15% yielded greater shear bond strength than the 0.03% concentration.
Thimmaiah et al. 2019 [30]	CPP-ACPF TCP nHAP No treatment (negative control)	Substrate: Enamel specimens were prepared from human premolars; Early artificial carious lesions were formed before treatment. Model: artificial saliva for 30 days. Treatment: treatments were applied daily using a cotton applicator tip. Assessment: mineral content by SEM-EDX.	Particle size: not reported. Format: Commercially available 1% nHAP (toothpaste). Aclaim^®^ toothpaste (Group pharmaceuticals Ltd., Mumbai, India).	The group treated with nHAP revealed lowest gain of Ca and P.
Hanafay et al. 2019 [47]	Chitosan hydrogel group Zinc doped nHAP	Substrate: Enamel specimens were prepared from human premolars; Early artificial carious lesions were formed before treatment. Model: 21 days in artificial saliva. Treatment: 2 × 2 min daily. Assessment: mineral content by SEM-EDX, crystallisation by XRD.	Particle shape/size: hexagonal particles with 15 nm diameter and 130 nm length. Format: Pure nHAP was manufactured by the wet-chemistry method into paste form.	Both groups were able to repair enamel as demonstrated by SEM analysis, which revealed absence of porosities and formation of apatite layer. Mineral recovery (Ca/P ratio) was evident in both test groups.
Vijayasankari et al. 2019 [15]	1% nHAP (commercial) 1% nHAP (experimental) 10% nHAP (experimental) CPP-ACP No treatment (Negative control)	Substrate: Enamel specimens were prepared from human primary molar; Early artificial carious lesions were formed before treatment. Model: 14 days in artificial saliva. Treatment: 2 × 3 min daily. Assessment: mineral content by SEM-EDX.	Particle size: not reported. Format: Experimental nHAP was synthesized using the sol-gel method. 1% and 10% concentrations were prepared in paste form. No size data reported.	A significant increase in Ca and P levels on the enamel surface treated with 10% nHAP as compared with other groups.
Manchery et al. 2019 [17]	nHAP 5% Calcium sodium phosphosilicate 1450 ppm Amine flouride	Substrate: Enamel specimens were prepared from human premolars; Early artificial carious lesions were formed before treatment. Model: pH cycling for 7 days. Treatment: 3 × 1 min daily. Assessment: lesion depth by polarized light microscope.	Particle size: not reported. Format: 10% nHAP was used (APAGARD ROYAL, Sangi Company, Tokyo, Japan) (toothpaste).	The lesion depth decreased significantly by 10.56% in nHAP group, 6.73% in 5% Calcium sodium phosphosilicate group, and 9.58% in Amine fluoride group.
Bossu et al. 2019 [14]	Common toothpaste with no active component Commercial fluoride toothpaste (500 ppm) Commercial fluoride toothpaste (1400 ppm) nHAP nanocrystal toothpaste	Substrate: Enamel specimens were prepared from human primary molar; Early artificial carious lesions were formed before treatment. Model: 15 days in saline solution. Treatment: specimens were brushed with the pastes, 3 × 2 min. Assessment: High Resolution SEM and Variable Pressure SEM.	Particle size: 50 to 100 nm. Format: nHAP was obtained in paste form. (Biorepair, Coswell SpA, Bologna, Italy).	Teeth treated with nHAP (Biorepair) toothpaste revealed uniform distribution of material while the surfaces treated with fluoride toothpaste revealed rough surfaces.
Joshi et al. 2019 [31]	Bioactive glass (Novamin) nHAP f-TCP Grape seed extract 1000 ppm fluoride toothpaste (positive control) Distilled water (negative control)	Substrate: Enamel specimens were prepared from human premolars; Early artificial carious lesions were formed before treatment. Model: pH cycling for 21 days. Treatment: 2 × 2 min daily. Assessment: surface microhardness by Vickers hardness test.	Particle size: not reported. Format: nHAP toothpaste was commercially obtained from Acclaim pharmaceutical, India. (toothpaste).	nHAP treated enamel showed similar surface micro-hardness recovery in contrast to bioactive glass and f-TCP. nHAP treated enamel demonstrated higher surface hardness recovery in contrast to grape seed extract and negative control.
Konagala et al., 2020 [38]	Arginine Fluoride varnish nHAP Arginine + Fluoride varnish Arginine + nHAP	Substrate: Enamel specimens were prepared from human maxillary incisors Model: Early artificial carious lesions were formed after treatment, followed by pH cycling for 10 days. Treatment: Twice daily. Assessment: surface microhardness by Vickers hardness test Vickers hardness test, mineral content by SEM-EDX.	Particle size: not reported. Format: 10% nHAP powder (Sigma Aldrich, St. Lous, MA, USA) (1 g nHAP + 10 mL distilled water) was used to prepare the samples.	Significantly lower surface micro-hardness value was revealed when nHAP was used alone. Arginine demonstrated to have an enhanced synergistic remineralization effect when used in combination with nHAP.
Xu et al. 2020 [32]	nHAP Zn substituted nHAP nHAP@Polyacrylic acid Zn substituted nHAP@Polyacrylic acid nHAP@ Alendronate-grafted polyacrylic acid Zn substituted nHAP@Alendronate-grafted polyacrylic acid Phosphate-buffered saline treatment (negative control)	Substrate: Enamel specimens were prepared from bovine incisors; Early artificial carious lesions were formed before treatment. Model: 14 days in artificial saliva. Treatment: One-off aqueous solutions were dropped on the demineralized enamel. Assessment: Surface microhardness by Knoop micro-hardness tester; surface morphology by SEM.	Particle size: Zn substituted nHA@ALN-PA (Diameter: 3–10 nm, Length: 70–110 nm). Format: Powder form of nHAP was obtained to synthesize Zn substituted nHA@ALN-PAA.	Enamel surface microhardness was significantly improved in all treatment groups compared to the negative control. Zn substituted HA showed superior enamel surface microhardness compared to the groups without Zn substitution.

AFM: Atomic force microscopy; APF: Acidulated phosphate fluoride; CPP-ACP: Casein Phosphopeptide-amorphous Calcium Phosphate; Ca: Calcium; nHAP: Hydroxyapatite nanoparticles; D%: Demineralization %, R%: Remineralization; FTIR: Fourier Transform Infrared Spectroscopy; NaF: Sodium Fluoride; P: Phosphorus; SEM: Scanning Electron Microscope; SEM-EDX: Scanning Electron Microscope and Energy dispersive X-ray analysis; TCP: Tri-calcium phosphate; f-TCP: Functionalized tri-calcium phosphate; Sr: Strontium; TEM: Transmission electron microscopy; XRD: X-ray diffraction; Zn: Zinc.

**Table 2:** Summary of the effect of hydroxyapatite nanoparticles on dentine/cementum mineralization.

Reference	Experimental Groups	Study Model and Design	nHAP Details	Main Findings on nHAP
Tschoppe et al., 2011 [20]	1.5 mM Calcium and phosphate 20 wt% Zinc carbonate-nHAP 24 wt% Zinc carbonate-nHAP 0.14 wt% Aminefluoride 7 wt% nHAP	Substrate: Dentine specimens were prepared from cervical regions of bovine teeth. Early artificial carious lesions were formed before treatment. Model: Remineralizing solution for 2 and 5 weeks. Treatment: 2 × 2 min daily. Assessment: Mineral loss and lesion depth by transverse microradiography.	Particle size: 20 nm in size, with granular dimensions up to 100–150 nm. Format: Commercially available nHAP in paste form (BioRepair and BioRepair Sensitive; Dr. Kurt Wolff, Bielefeld, Germany) were obtained and diluted to form a slurry homogenous mixture.	The dentine treated with nHAP exhibited significantly less mineral loss and lesion depth when compared with dentine treated with amineflouride after 5 weeks.
Comar et al., 2013 [19]	20% nHAP 20% nHAP+ 0.2% NaF 10% nHAP 10% nHAP+ 0.2% NaF Placebo paste (without fluoride and nHAP) 0.2% NaF CPP-ACP CPP-ACP+ 0.2% NaF	Substrate: Root dentine specimens were prepared from bovine teeth. Model: pH cycling for 7 days. Treatment: Brushed for 1 min, twice a day. Assessment: Surface microhardness by Knoop hardness tests.	Particle size: Experimental paste consisted of calcium phosphate (100 nm). Format: paste form.	nHAP treatment was unable to reduce the loss of dentine surface hardness regardless of fluoride addition.
Besinis et al., 2014 [44]	Sound dentine (control 1) Non-infiltrated dentine matrix (control 2) nHAP control Experimental nHAP nSiO_2_	Substrate: Dentine specimens were prepared from human premolars, and completely demineralized to dentine collagen matrix. Model: four weeks in artificial saliva. Treatment: treatment infiltration for 24 h. Assessment: mineral recovery by micro-CT, collagen morphology and elemental analysis by TEM and EDX.	Particle size: control nHAP 27.23 ± 23.96 nm, Experimental nHA 3.51 ± 0.87 nm. Format: n-HAP control: commercially synthesized obtained from plasma Biotal Ltd., Buxton, UK. Experimental n-HAP: (synthesized by the research group following the sol–gel technique, 15% (*w/v*).	nHAP treatment increased Ca and P levels of the local scaffolds of dentine matrix. nHAP treatment was unable to recover the minerals from the dentine collagen matrix, however, it reduced the porosity of the dentine collagen matrix.
Iijma et al., 2019 [42]	No treatment (Negative control) 0% S-PRG filler paste 5% S-PRG filler paste 30% S-PRG filler paste nHAP paste	Substrate: Dentine specimens were prepared from human premolars. Early artificial carious lesions were formed before treatment. Model: remineralization solution (artificial saliva) for 1 month. Treatment: pastes were polished for 10 s weekly. Assessment: Surface microhardness by nano-indentation tests, surface morphology by SEM.	Particle size: not reported. Format: nHAP paste was commercially obtained from Renamel/Sangi brand (toothpaste).	Dentine surface treated with S-PGR paste showed higher nano-hardness in contrast to nHAP treated surface. Dentine surface treated with nHAP have partly filled the dentinal tubules in contrast to that treated with S-PGR filler paste.
Juntavee et al., 2018 [41]	nHAP gel 0.21% NaF: (Clinpro) Deionized water (negative control)	Substrate: Cementum specimens were prepared from human mandibular third molars; Early artificial carious lesions were formed before treatment. Model: Remineralized gels for 30 days. Treatment: 2 × 4 min daily. Assessment: Surface microhardness by Vickers Microhardness, surface morphology by SEM.	Particle size: not reported. Format: Gel form of nHAP was obtained (10% nano-hydroxymethyl cellulose).	Hardness values revealed a significantly greater hardness of nHAP in contrast to NaF treatment. Generalized irregularities of demineralized cementum with opening tubules also indicated the deposition of nHAP.
Leal et al., 2020 [43]	Placebo (toothpaste without fluoride and nHAP) nHAP 1100 μg F/g 1100 μg F/g + nHAP 5000 μg F/g 5000 μg F/g + nHAP	Substrate: Dentine specimens were obtained from bovine incisors. Model: pH cycling for 10 days. Treatment: Twice a day for 5 min. Assessment: Cross-sectional microhardness up to 200 μm from lesion surface.	Particle size: ranging from 0.83 to 3.85 nm. Format: 20% nHAP toothpaste.	nHAP when used in combination with 5000 μgF/g, showed a smaller dentine lesion as compared to the placebo group.

Ca: Calcium; CPP-ACP: Casein phosphopeptide-amorphous calcium phosphate; EDX: Energy-dispersive X-ray spectroscopy; F: Fluoride; micro-CT: Micro-Computed tomography; MTT: 3-(4,5-dimethylthiazol-2-yl)-2,5-diphenyltetrazolium bromide cell viability assay; NaF: Sodium fluoride; nHAP: Hydroxyapatite nanoparticles; P: Phosphorus; SEM: Scanning electron microscope; SiO_2_: Silicon dioxide; S-PRG: Surface reaction type pre-reacted glass ionomer filler; TEM: Transmission electron microscopy.

**Table 3:** Summary of the antimicrobial effect of hydroxyapatite nanoparticles.

Reference	Experimental Groups	Study Model and Design	nHAP Details	Main Findings on nHAP
Park et al. 2019 [45]	5% nHAP 1% Sucrose 5% nHAP with 1% sucrose No treatment (negative control)	Bacterial species *Streptococcus mutans.* Model: *Streptococcus mutans* were incubated overnight in either brain heart infusion or basal medium mucin and exposed to the experimental groups. Incubation: 24 h. Assessment: cell survival by colony-forming unit and MTT staining.	Particle size: (20–50 nm; 5–10% *w/v*). Format: nHAP were commercially obtained (Alfa Aesar sol).	nHAP enhanced biofilm formation in the presence of sucrose by increasing glucosyltransferase transcription.
Xu et al. 2020 [32]	nHAP Zn substituted nHAP nHAP@Polyacrylic acid Zn substituted nHAP@Polyacrylic acid nHAP@ Alendronate-grafted polyacrylic acid Zn substituted nHAP@Alendronate-grafted polyacrylic acid Phosphate-buffered saline treatment (negative control)	Bacterial species *Streptococcus mutans*. Substrate: Bovine enamel specimens were cultured with *Streptococcus mutants*. Model: Bacterial suspensions were cultured with 40 mg nanomaterials (experimental groups). Incubation: 12 h at 37 °C. Assessment: pH, cell viability by colony-forming unit and Fourier-transform infrared spectroscopy.	Particle size: Zn substituted nHAP @ALN-PAA (Diameter: 3–10 nm, Length: 70–110 nm). Format: Powder form of nHAP was obtained to synthesize Zn substituted nHA @ALN-PAA.	Significant improvement in antibacterial activity against *Streptococcus mutans* was demonstrated in Zn substituted nHAP groups.
Ionescu et al. 2020 [46]	Zn-carbonate substituted nHAP F, Mg, Sr-carbonate substituted nHAP Distilled water (negative control)	Bacterial species: *Streptococcus mutans* biofilm and artificial oral microcosm based on mixed oral flora. Substrate: human enamel blocks and resin-based composites were used to develop biofilm of oral microcosm and *Streptococcus mutans*. Treatment: samples were brushed with pastes for 2 min, rinsed, and sterilized prior to microbiological assessment. Model: Shaking microtiter plate and modified drip flow bioreactor was utilized to mimic salivary conditions. Incubation time: 12 h and 24 h at 37 °C. Assessment: biofilm morphology and elemental content by SEM-EDX, cell viability by MTT assay and confocal microscopy.	Particle size: not reported. Format: The toothpastes were commercially obtained from A: Biorepair total protection plus (Coswell Funo, Italy) and B: Biosmalto Caries (Curasept, Italy).	The biofilm treated with F, Mg, Sr-carbonate substituted nHAP demonstrated lower values of early colonisation in comparison with the biofilm treated with Zn-carbonate substituted nHAP.

F: Fluoride; IR: Inhibition ratio; Mg: Magnesium; MTT: 3-(4,5-dimethylthiazol-2-yl)-2,5-diphenyltetrazolium bromide Cell viability assay; nHAP: Hydroxyapatite nanoparticles; Sr: Strontium; Zn: Zinc.

## 4. Discussion

While a recent systematic review, which was based on in vivo and in situ studies of nHAP in caries prevention, has been undertaken with a proper risk of bias evaluation [10], only four studies were included in the analysis. The major reasons for exclusion of studies were lack of a control group, a short study duration (less than 1 month), and not reporting an Institutional Review Board (IRB) approval. This very low number of included studies resulted in inconclusive evidence for the efficacy of nHAP. This highlights the need for more well controlled clinical trials to be undertaken in the future. Ideally, a risk of bias of the included studies should also be provided in the current review. In particular, studies that provide no details of the nHAP particle size and concentrations compromise the quality of the review.

A wide range of nHAP structures, such as nano-rods, microspheres, or hierarchically nano-structures, are reported in the articles reviewed here, and these potentially influence the effects exerted by nHAP. Indeed, it has been previously demonstrated that the morphology of the nanoparticles will affect the speed of Ca^2+^ release, as Ca^2+^ in microspheres appears to release more rapidly than hierarchically nano-structures [48]. The synthesized nHAP in the reviewed studies was prepared by using several different techniques, including a hydrothermal method, a sol-gel method, and a wet-chemistry method, which are believed to be able to provide high phase purity and grain sizes of between 20–50 nm [49]. Aqueous nHAP slurry in distilled water provides a relatively simple approach to assess the effect of the nanoparticles on the caries lesion. However, it may not be highly clinically relevant. Furthermore, nHAP was incorporated into several different formats of dental products, including toothpastes, creams, and restorative materials. Studies used either commercialized dental products, which contained nHAP, or incorporated nHAP into existing toothpastes by a certain weight percentage. One study attempted to incorporate nHAP into the resin infiltration, and they found that this test material caused higher enamel resistance against demineralization compared with the control nHAP-free infiltration [39].

The most common substrate used in the studies was enamel with chemically-induced artificial early caries to mimic the clinical situation of incipient caries (non-cavitied) presented on the coronal side of the tooth. The human enamel includes morphologically aligned, parallel, up to 70 nm in width and 30 nm in thickness, micron-long carbonated hydroxyapatite nanocrystals, bundled either into 5-μm-wide rods (prisms) or a space-filling interrod arrangement [50]. When caries (demineralization) are initiated, the enamel prism junctions are enlarged and destruction of the interprismatic structure is observed [51], while the size of nHAP enhances its penetration into the interprismatic enamel space [52]. Moreover, a few matrix proteins remain embedded in the enamel’s organic component and these proteins operate as a scaffold for conduction of nHAPs so they can be easily deposited within the nano-gaps [53]. These proteins enable the capture of the minerals and subsequently facilitate mineral apatite formation [52]. In comparison, fluoride has been shown to predominately integrate into the surface of the initial caries lesion, while being less effective in deep sub-surface lesions and this effect has been found alongside both high fluoride and low fluoride treatment [54]. This lamination effect results in a limitation in the dose-dependent effectiveness of fluoride products, therefore, creating a negative effect for complete remineralization into the deeper zones and subsequently limiting application [55]. An in situ study, which compared the effectiveness of two toothpastes containing nHAP or fluoride in promoting remineralization of initial enamel caries, found that while nHAP achieved comparable efficacy with 500 ppm fluoride, it enabled a more homogenous lesion of remineralization when compared with the fluoride induced lesion surface lamination following transverse microradiography analysis [56]. Therefore, nHAP should be developed and applied to enhance the effect of existing fluoride therapies rather than to replace them, but it still provides an alternative option to those who are reluctant to use fluoride.

Root dentine rather than cementum is the most frequently used substrate when investigating root caries. This is due to the cementum being relatively thin (20 to 50 microns near the cemento-enamel junction) [57]. Therefore, the dentine beneath the cementum is considered the major component in root caries progression. The results of the studies on dentine are somewhat inconsistent, which might be due to the different experimental conditions and different particle sizes used. Comar et al. [19] found that an nHAP paste was unable to reduce the loss of dentine surface hardness, which may be due to the relatively large size of the nHAP particle used in their study, which was 100 nm and already reached the maximum size due to the definition of a nanomaterial. It is plausible that a mechanical imbrication of the paste into the inter-tubular dentine space due to the size of the particles occurred. Besinis et al. [44] reduced fully demineralized dentine blocks to a collagen matrix prior to the nHAP treatment. They found that the nHAP with a particles size of ~20 nm was able to locally infiltrate into the scaffolds with increased levels of calcium and phosphate deposition. Both Tschoppe et al. [20] and Leal et al. [43] demonstrated an increased remineralizing effect on dentine when nHAP was used alone or with fluoride; the particles sizes of their studies were less than 20 nm. Juntavee et al. [41] showed that nHAP containing gels had a higher capability for the remineralization of cementum when compared with fluoride varnish, which was possibly associated with the nanoparticle size as it was capable of constructive interdigitation with the cementum structure. Future studies should focus on the mechanisms of nHAP-induced remineralization at a more in-depth level to determine the role of nHAP in the calcium phosphate formation process and to determine how the size of the nHAP affects this process.

The antimicrobial effect of nHAP was negligeable given its high biocompatibility and low toxicity. In addition, it was also found that the formation of *S. mutan* biofilm was enhanced when *S. mutans* was co-cultured with the presence of 5% nHAP suspension [45]. Caries result from an ecological imbalance in the physiological equilibrium between oral microbial biofilms and tooth minerals. Maintaining tooth minerals and controlling oral microbial biofilms are essential for the control of caries [58]. Aimed at a dual action effect nHAP in both mineralization and antimicrobial effect, studies attempted to develop a hybrid nHAP by substituting calcium ions with metallic ions. Silver is a well-known antimicrobial agent due to its broad spectrum, low toxicity, and lack of cross-spectrum bacterial resistance [59]. Silver can be incorporated into the crystal structure of hydroxyapatite to produce silver-containing hydroxyapatite [60] to give a formula of Ca_10−x_Ag_x_(PO4)_6_(OH)_2_ with 0.0 ≤ x ≤ 0.5. Notably, a relatively small number of calcium ions are substituted with silver ions [61,62]. This silver-containing hydroxyapatite has been shown to reduce bacterial adhesion and to have minimal tissue cytotoxicity [60], however, aesthetic effects may be compromised due to the fact that silver has a black coloration when it is oxidized, which readily occurs in air [63]. Zinc is another commonly used metal which can substitute for the calcium ion in nHAP. In the orthopaedic field, it is regarded as an essential trace element that promotes bone formation and exhibits antimicrobial effects [3]. Several studies involving Zn-substituted apatite containing a low amount of zinc (<1 wt%) have been reported, and demonstrate the material to exhibit good bioactivity with antibacterial properties [64,65,66]. Indeed, Chung et al. [67] and Stanic et al. [66] observed a reduction of bacterial strains *Escherichia coli* (*E. coli*), *Staphylococcus aureus* (*S. aureus*), *Candida albicans* (*C. albicans*), and *S. mutans* cultured on Zn-substituted HAP. Furthermore, strontium is a divalent cation that is located in the same column of the periodic table as calcium. Consequently, strontium has chemical properties that are similar to those of calcium, and it can partly substitute for calcium and be incorporated into the crystal lattice structure of hydroxyapatite. Sr-substituted HAP also demonstrates good bioactivity and can directly bond to bony tissue under non-weight-bearing conditions [68]. A synergistic effect of strontium and fluoride on hydroxyapatite formation has been demonstrated in previous studies, and strontium was able to aid remineralization of a carious lesion in the presence of fluoride [69,70]. Sr-substituted nHAP has been shown to display enhanced biocompatibility compared with pure nHAP [26]. However, reports on the application of nanosized metal-substituted HAP in caries management are limited. One previous study found that the inhibition ratio of synthesized Zn-substituted nHAP against *S. mutans* was significantly higher compared with the pure nHAP group [32]. A further study used a toothpaste containing Mg-Sr-F substituted hydroxyapatite and demonstrated that it exerted a decreased rate of early colonisation of *S. mutans* in comparison with those containing Zn-substituted nHAP after 12 h. This may be due to the synergistic effect of fluoride and strontium in caries management [46,69,70]. Notably, instead of calcium being substituted by metal cations, fluorohydroxyapatite (Ca_5_(PO_4_)_3_(OH)_1−x_F_x_) could be formed by exchanging fluoride ions for the hydroxyl group in the HAP; the isotropic distribution of the charge on fluoride anions allows for a better fit in the lattice structure compared with the larger asymmetric hydroxyl ions. This produces a more ordered apatite structure, which is characterized by increased thermal and chemical stability compared with HAP [63,71]. Future studies on metal-substituted fluorohydroxyapatite nanoparticles could identify this as a promising synergistic strategy for caries management.

## 5. Conclusions

This review of the current literature concludes that nHAP can promote mineralization in early caries lesions, potentially by being incorporated into the porous tooth structure caused by the caries process and thereby increasing its mineral content and hardness. The particle size of nHAP plays an important role in this remineralization process. The antimicrobial effect of nHAP can be potentially achieved by using metal substituted nHAP. All the above properties highlight nHAP as a promising bioactive material for potential use in the management of dental caries, which should be explored in further clinical research.

## Data Availability

Not applicable.
